# p31^comet^-Induced Cell Death Is Mediated by Binding and Inactivation of Mad2

**DOI:** 10.1371/journal.pone.0141523

**Published:** 2015-11-06

**Authors:** Hyun-Jin Shin, Eun-Ran Park, Sun-Hee Yun, Su-Hyeon Kim, Won-Hee Jung, Seon Rang Woo, Hyun-Yoo Joo, Su Hwa Jang, Hee Yong Chung, Sung Hee Hong, Myung-Haing Cho, Joong-Jean Park, Miyong Yun, Kee-Ho Lee

**Affiliations:** 1 Division of Radiation Cancer Research, Korea Institute of Radiological & Medical Sciences, Seoul 139–706, Korea; 2 Graduate School of Biomedical Science and Engineering, Hanyang University, Seoul 133–791, Korea; 3 Laboratory of Toxicology, College of Veterinary Medicine, Seoul National University, Seoul 151-74-2, Korea; 4 Department of Physiology, College of Medicine, Korea University, Seoul 136–705, Korea; 5 College of Oriental Medicine, Kyung Hee University, 1 Hoegi-dong, Dongdaemun-gu, Seoul 130–701, Korea; 6 Department of Pathology and BK 21 PLUS Project for Medical Science, Yonsei University College of Medicine, Seoul, 120–749, Korea; University of Edinburgh, UNITED KINGDOM

## Abstract

Mad2, a key component of the spindle checkpoint, is closely associated with chromosomal instability and poor prognosis in cancer. p31^comet^ is a Mad2-interacting protein that serves as a spindle checkpoint silencer at mitosis. In this study, we showed that p31^comet^-induced apoptosis and senescence occur *via* counteraction of Mad2 activity. Upon retroviral transduction of p31^comet^, the majority of human cancer cell lines tested lost the ability to form colonies in a low-density seeding assay. Cancer cells with p31^comet^ overexpression underwent distinct apoptosis and/or senescence, irrespective of p53 status, confirming the cytotoxicity of p31^comet^. Interestingly, both cytotoxic and Mad2 binding activities were eliminated upon deletion of the C-terminal 30 amino acids of p31^comet^. Point mutation or deletion of the region affecting Mad2 binding additionally abolished cytotoxic activity. Consistently, wild-type Mad2 interacting with p31^comet^, but not its non-binding mutant, inhibited cell death, indicating that the mechanism of p31^comet^-induced cell death involves Mad2 inactivation. Our results clearly suggest that the regions of p31^comet^ affecting interactions with Mad2, including the C-terminus, are essential for induction of cell death. The finding that p31^comet^-induced cell death is mediated by interactions with Mad2 that lead to its inactivation is potentially applicable in anticancer therapy.

## Introduction

The spindle checkpoint controls the activity of anaphase-promoting complex/cyclosome (APC/C), a E3 ubiquitin ligase that destroys chromosomal cohesion and subsequently causes sister chromatid separation and mitotic exit. Until all sister chromatids are appropriately attached to the spindle, the checkpoint serves to inhibit APC/C, thereby delaying anaphase onset and ensuring accurate chromosomal segregation [1]. Impairment of the spindle checkpoint allows cells to progress through mitosis, even when chromosomal segregation is not completed. Generation of aneuploid chromosomes leads to various forms of chromosomal instability [2]. Such abnormalities are part of the initial phenotype of human cancers and serve as hallmarks of cancer development. The appearance of aberrations can therefore be used to predict clinical outcomes [3–5]. Several mutations and aberrant expression of spindle checkpoint regulators in human cancers have been defined to date [6–10].

Mad2, a key component of the spindle checkpoint, is frequently overexpressed in a variety of human cancers, including colorectal [10], lung [11], and liver cancer [12], and soft tissue sarcoma [13]. Aberrant Mad2 expression promotes chromosomal instability, in turn, inducing tumorigenesis and relapse in mice [14]. Mad2 expression is associated with susceptibility to anticancer drugs [15] and prognosis of cancer patients [10, 11]. Recently, a Mad2-interacting protein, p31^comet^, was characterized as a spindle checkpoint silencer at mitosis [16–21]. The protein is a structural mimic of O-Mad2 (open form) and binds to C-Mad2 (closed form). This binding prevents dimerization of Mad2, in turn, inhibiting the conformational change of O-Mad2 to C-Mad2, the active form, and triggering exit from mitotic checkpoint arrest [16–18]. An earlier report showed that p31^comet^ directly promotes disassembly of the mitotic checkpoint complex [22]. Another recent study demonstrated that p31^comet^-induced inhibition of Mad2 conformational changes prior to disassembly is the mechanistic basis of spindle checkpoint silencing.

Based on the suggestion that Mad2 exerts oncogenic activity [10–14, 23], we hypothesized that abnormal control of Mad2 function affects cell survival by inducing chromosomal instability, accompanied by chromosomal missegregation and development of aneuploidy. Therefore, inactivation of Mad2 may present a promising anticancer therapeutic strategy. Previously, we reported that p31^comet^ overexpression leads to senescence and apoptosis in cancer cells [24]. However, the underlying mechanism of p31^comet^-induced death and its structural association with Mad2 remain to be established. In the current study, we analyzed the effects of sequence deletion of p31^comet^ on cell survival to determine the region critical for cytotoxic activity and potential association of cell death induction with Mad2. Our data clearly indicate that overexpressed p31^comet^ induces apoptosis and senescence, leading to suppression of clonal survival of cancer cell lines. Furthermore, this cytotoxic activity of p31^comet^ is mediated through binding and inactivation of Mad2. Additional regions that influence Mad2 interactions, including the C-terminus, are also important for p31^comet^-mediated cell death.

## Materials and Methods

### Cell culture and reagents

To evaluate the cytotoxic effects of p31^comet^, human tumor cell lines with different tissue origins (lung cancer A549, Calu-1, SK-Lu-1, H460, H446, H1299; cervical cancer HeLa, SW756, SiHa, MS751, Caski; liver cancer Sk-HEP-1, Chang, Hep3B, Huh-7, HepG2; osteosarcoma Saos-2, U2OS; breast cancer MCF-7) were used. In addition, WI38, MRC5, and BJT fibroblasts were employed for evaluation of normal cell response to p31^comet^ overexpression. Cell lines were purchased from ATCC (American Type Culture Collection, Manassas, VA) and grown in the recommended media supplemented with 10% FBS and 1% penicillin-streptomycin at 37°C in 5% CO_2_.

Mouse embryonic fibroblasts (MEF) and NIH3T3 cells were maintained in Dulbecco’s Modified Eagle’s Medium supplemented with 10% (v/v) fetal bovine serum and the appropriate antibiotics. *p53-/-* MEFs were prepared by intercrossing heterozygote mice from the Jackson Laboratory. *INK4a-/-* MEFs were kindly provided by Dr. R. A. Depinho [25].

### Plasmid and siRNA construction

Human and mouse *p31* ^comet^ and *Mad2* cDNA were cloned into the MFG retroviral vector as described previously [24]. Briefly, cDNAs encoding human *p31* ^comet^, mouse *p31* ^comet^ and *Mad2* were amplified *via* PCR and cloned into *MFG-IRES-puro* and *MFG-IRES-EGFP* vectors between the Not I and PAC I restriction sites. Deletion constructs of human *p31* ^comet^ were similarly amplified using PCR and cloned within the same restriction sites. The *p31ΔN15*, *p31ΔN54*, *p31ΔN64*, *p31ΔC30*, *p31ΔC60*, *p31ΔC120*, and *p31ΔC170* constructs were prepared by deleting 15, 54 or 64 amino acids from the N-terminus and 30, 60, 120 or 170 amino acids from the C-terminus, respectively. *p31ΔM2*, depleted of the site affecting Mad2 binding activity, was previously generated *via* site-directed mutagenesis [24]. The *p31Q83A/F191A* mutant defective in Mad2 binding [17] was additionally generated *via* site-directed mutagenesis using *pCIN-p31* and *MFG-IRES-puro-p31* as templates. The double mutant was sequentially constructed using the complementary primer set, 5′-aagcatatcatgtatgcacgccagcagctccct-3′ and 5′-tgtttgcgccgtctcgcccgagccatattcatg-3′. The dominant-negative mutant of *Mad2 (R133E/Q134A)* defective in binding to p31^comet^ was kindly provided by Dr. A. Musacchio [18]. The siRNA oligonucleotide sequences were as follows: si-control (5’-ACGUGACACGUUCGGAGAAUU-3’) and si-*Mad2* (5’-UCCGUUCAGUGAUCAGACA-3’). siRNAs were transfected into cells using RNAiMAX (Invitrogen, Carlsbad, CA), according to the manufacturer's protocol.

### Retrovirus infection

Recombinant retroviruses were produced using H29D or 293T cells. Cloned human *p31* ^comet^, mouse *p31* ^comet^, human *Mad2* and empty *pMFG-EGFP* vectors were transfected using Lipofectamine into H29D cells or co-transfected with plasmids encoding the retroviral structure proteins, pGag-Pol and pVSV-G, into 293T cells using PEI [polyethylenimine] (Polysciences Inc, Warrington, PA). After 24–72 h, the supernatant fractions of H29D or 293T cells were collected and filtered, and polybrene added at a concentration of 6–8 μg/ml. Retrovirus titers were determined by measuring GFP-positive colony formation using a NIH3T3 cell line. Cells were retrovirally infected over 3–5 h. This step was repeated to increase the infection rate. After change of medium, growth of infected cells was monitored by enumeration of cells.

### Colony forming assay

Various human cancer cell lines were infected with retrovirus expressing p31^comet^. One day after infection, cells were plated at a density of 1.5 x 10^3^ cells in 100 mm dishes and incubated until the appearance of visible colonies. GFP-positive and -negative colonies were counted under a fluorescence microscope.

To assay colony formation by transformed foci, *p53-/- or INK4a-/-* MEFs within five passages or NIH3T3 cells were co-transfected with 2 μg plasmid encoding *c-myc*, *H-RASV12* [36] and test plasmids, including p31^comet^. Cells were plated at a density of 7 x10^5^ cells per 100 mm dish and transfected one day later using the calcium phosphate precipitation technique. At 10 to 12 days after transfection, colonies were visually counted after staining with crystal violet.

### Apoptosis and senescence assays

To detect apoptotic death, cells infected with retrovirus containing *p31* ^comet^, *Mad2* and empty vector plasmids were stained with APC-conjugated annexin V antibody (BD Biosciences, Franklin, NJ), in keeping with the manufacturer’s instructions. Fluorescence signals were quantitatively analyzed using flow cytometry (FACSCalibur, BD Biosciences). GFP-positive cells selected using the FL1 channel were gated, and 10,000 cells analyzed for Annexin V signal using the FL4 channel.

Senescence was detected by staining the cells with β-galactosidase activity [24]. Briefly, cells were fixed in 2% formaldehyde and 0.2% glutaraldehyde in PBS and stained with X-gal solution (1 mg/ml X-gal, 40 mmol citric acid/sodium phosphate, pH 6.0, 5 mmol potassium ferricyanide, 5 mmol ferrocyanide, 150 mmol NaCl, 2 mmol MgCl_2_). At 12 to 16 h later, X-gal stained cells were visualized under a light microscope.

### Transfection and immunoblotting

Cloned plasmids were transfected with the calcium phosphate co-precipitation method. At 48 h after transfection, cells were lysed with NP-40 lysis buffer (0.5% NP-40, 150 mM NaCl, 50 mM Tris, pH7.7) supplemented with protease inhibitors (Roche, Germany). Purified proteins were separated using SDS-PAGE and transferred to nitrocellulose membranes. Immunoblotting was performed using anti-p31^comet^ (ab150363, Abcam, Cambridge) and anti-β-actin (sc-134381, Santa Cruz Biotechnology, CA) or anti-Mad2 antibody (Bethyl Laboratories, Montgomery, TX). Reactive proteins were detected *via* development of chemiluminescence (Santa Cruz Biotechnology).

Immunoprecipitation was utilized to explore the interactions between Mad2 and human p31^comet^. Briefly, plasmid *PCI-neo-Myc-Mad2* and either wild-type or mutant *MFG-IRES-puro-Flag-p31* ^*comet*^ were transiently co-transfected into 293T cells with Turbofect (Fermentas Life Science, Ontario, Canada). Individual empty plasmids (*PCI-neo* and *MFG-IRES-puro*) served as negative controls. After 36 h of incubation, transfected cells were lysed with NP40-containing lysis buffer supplemented with protease inhibitors. FLAG-tagged proteins in cell lysates were immunoprecipitated with 0.5 μg anti-FLAG antibody (M2, Sigma). Mad2 protein in immunoprecipitates was detected using an anti-Myc antibody (2278, Cell Signaling, MA).

### Real-time RT-PCR analysis

mRNA expression in cells transduced with *p31* ^*comet*^ and empty vector was quantitatively analyzed with real-time RT-PCR. Total RNA was extracted using the Qiagen RNeasy Mini Kit (Qiagen, Valencia, CA) with the aid of DNase to eliminate cDNA contamination due to retrovirus, and quantity and quality monitored using a Nano Drop ND-1000 spectrophotometer (NanoDrop Technologies, Wilmington, Delaware) & Agilent 2100 Bioanalyzer (Agilent Technologies, Santa Clara, CA). Reverse transcription of template total RNA was performed with the iScript cDNA synthesis kit (Bio-Rad, Hercules, CA), and the synthesized cDNA amplified *via* RT-PCR with the Maxime PCR PreMix Kit (iNtRON Biotechnology, Kyungi-do, Korea). The following primer sequences were employed for real-time RT-PCR: *p31* ^comet^, 5`-CCG AAA ACC TTC TCC CCA G-3`(sense), 5`-GGT ACT AGT GTC CGT GCA AAG-3`(antisense); *β-actin*, 5`- ACC ACA CCT TCT ACA ATG AGC-3 (sense), 5`-CTT CAT GAT GGA GTT GAA GGT-3`(antisense).

### Cell cycle analysis

The cell cycle was analyzed in HeLa cells transduced with *Mad2* or control siRNA and *p31* ^comet^ or empty plasmid. At least 1 and 2 days after transduction, respectively, cells were treated with nocodazole (Sigma) at a concentration of 100 nM, fixed with ethanol, incubated with RNase, and subsequently stained with 50 ng/ml propidium iodide (Sigma). Changes in the cell cycle profile were analyzed using flow cytometry (FACSCalibur, BD Biosciences).

## Results

### p31^comet^ inhibits clonal survival in a broad range of human cancer cell lines

Previously, we showed that p31^comet^ overexpression leads to both senescence and apoptosis of human cancer cell lines [24]. To confirm and extend these observations, survival levels of various cancer cell lines were examined after retroviral transduction of human *p31* ^comet^- or GFP-encoding empty control vector. Retroviral transduction of *p31* ^comet^ has been shown to induce protein expression to a significant extent in human cancer cell lines [24]. In our experiment, transduction of empty vector yielded several GFP-positive colonies in numbers proportional to the vector infectivity level whereas *p31* ^comet^ induced a marked reduction in GFP-positive colony formation (Table 1). Among the 19 cancer cell lines derived from six different organs (lung, cervix, breast, liver, bone and kidney), 17 did not yield visible GFP-positive colonies, even when GFP-negative colonies were apparent, indicating that cancer cells overexpressing p31^comet^ cannot clonally survive for a length of time that permits cell division to occur. The H460 and HepG2 cell lines yielded GFP-positive colonies, but cell survival rates were only 16.4% and 24.8%, respectively, as calculated by dividing the number of GFP-positive colonies from cells encoding *p31* ^*comet*^ with that from cells containing empty vector. This finding indicates that some populations of cell lines can overcome cytotoxicity mediated by p31^comet^. However, in general, overexpression of p31^comet^ results in inhibition of clonal survival in a broad range of human cancer cell lines.

**Table 1:** p31comet is cytotoxic in a variety of cancer cell lines.

	hp31^comet^			Control		
	Colony No.		Infectivity	Colony No.		Infectivity
	GFP(+)	Total	(%)	GFP(+)	Total	(%)
**Lung**						
Calu-1	0	0.3	93	226.3	252	95.5
SK-Lu-1	0	34.7	72.1	189.7	197.7	80.2
H460	0	2.7	88	172	187	86
H446	25	45.7	85.4	152	178.3	63.6
	0	0	93	154.7	160.3	95.3
**Cervix**						
Hela	0	2.3	87.2	242.7	285	86.3
SW756	0	155.7	48.2	186.3	299	66.7
SiHa	0	2.7	67.2	194.7	283.7	67.2
MS751	0	37	52.3	108.3	172	53.4
Caski	0	2.7	84.3	159	165.2	84.9
**Breast**						
MCF-7	0	155.9	45.5	270.3	385.3	75.4
**Liver**						
Sk-Hep-1	0	148.7	57.9	115	195	53.3
Chang	0	42	73.7	177	248.3	85.6
Hep3B	0	143.7	48.4	61	235.7	35.7
Huh-7	0	17.7	67.9	61.3	96.7	73.3
HepG2	44.7	70	89.3	180	196.7	85.3
**Bone**						
SaOs-2	0	126.7	51.2	120.7	268	56.8
U-2OS	0	2.3	91.7	188.7	200.3	86.2
**Kidney**						
293	0	29.7	82.4	193.3	243	80.6

Nineteen human cancer cell lines were infected with retroviruses containing hp31comet-IRES-EGFP or empty control IRES-EGFP plasmids. The numbers of resulting GFP-positive and -negative colonies were counted. The totals reflect the sums of such colonies. Each infectivity value is a percentage of the number of GFP-positive cells that underwent retroviral transduction.

### p31^comet^ overexpression leads to apoptosis and senescence, irrespective of p53 status

**Fig 1:**
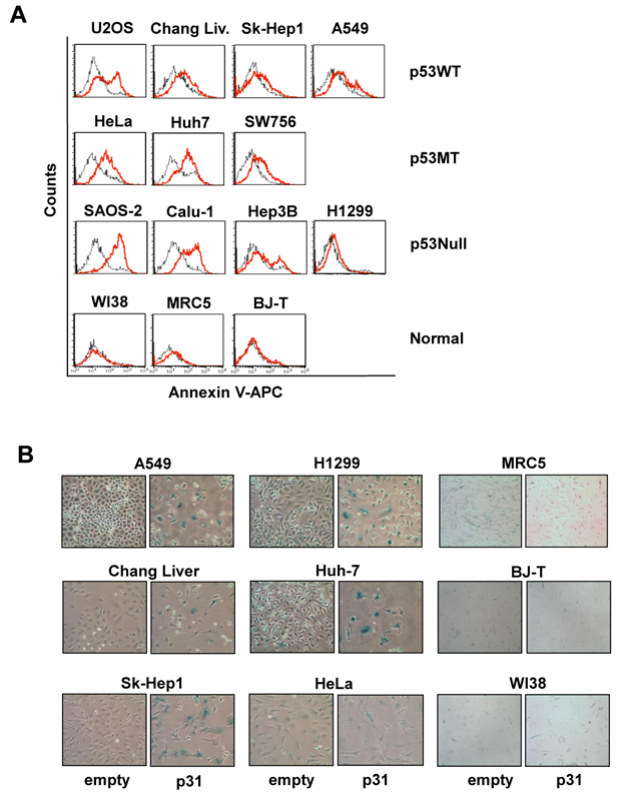
p31^comet^ induces apoptosis and/or senescence in various cancer cell lines. Various human cancer and normal cell lines were transfected with retroviruses containing *p31* ^comet^ *-IRES-EGFP* or *IRES-EGFP* control plasmids. (A) At 5 days after retroviral infection, apoptosis was analyzed with a flow cytometry histogram of Annexin V staining in 10 cancer and 3 normal cell lines. Only infected cells with GFP fluorescence were analyzed *via* gating, and the histograms between cells transduced with *p31* ^comet^ (red line) and empty vector (black line) overlapped for analysis. (B) At 5 days after retroviral infection of *p31* ^comet^ or empty vector, senescence determined based on β-gal staining was observed using light microscopy (x 100; except MRC5, x 40).

To further determine whether death of the cancer cell lines examined occurred *via* cellular apoptosis and senescence, the two distinct phenotypes reported previously by our group [24], these processes were analyzed in 11 cancer cell lines, including those used in the cited report (A549 cells primarily undergo senescence while HeLa cells display both phenotypes). In the earlier investigation, A549 cells expressing wt p53 underwent p31^comet^-induced senescence. Therefore, cancer cell lines with evident p53 status were selected to ascertain whether p53 status affects phenotypes. As shown previously [24], our data confirmed that HeLa cells exhibit distinctly higher Annexin V fluorescence upon retroviral infection with *p31* ^comet^, whereas A549 cells exhibit only slightly higher fluorescence, compared to infection with empty vector (Fig 1A). In extended analysis of the other nine cancer cell lines, increase in Annexin V staining upon retroviral infection was detected not only in cancer cell lines defective in p53 function, either due to deletion (Saos-2 [26] and Calu-1 [27]), point mutation (Huh7 cells [28]) or human papilloma virus infection (SW756 [29]), but also in those with wild-type p53 (U2OS [30], Chang liver [31], and SK-Hep1 [32]). Our results clearly indicate that retroviral transduction of *p31* ^comet^ induces apoptosis in cancer cell lines, irrespective of p53 status. Next, we examined senescence by staining adherent cells with β-galactosidase (β-gal) after eliminating floating cells. As with apoptosis, senescence detected with β-gal staining was also evident in cells retrovirally infected with *p31* ^comet^, regardless of p53 status (Fig 1B). Cells displaying both distinct (HeLa, Huh7, Chang liver) and weak (A549 and H1299) Annexin staining exhibited senescence-associated β-gal staining. These β-gal stained cells displayed flat and enlarged morphology under light microscopy (Fig 1B). The remnant adherent cells did not form colonies (Table 1), implying mitotically inactive death. As shown previously [24], the cancer cell lines infected with *p31* ^comet^ displayed elevated levels of translated protein, compared to those infected with empty vector (S1 Fig). Our results indicate that p31^comet^ overexpression leads to apoptosis and/or senescence in cancer cells, irrespective of p53 status.

To explore the effects of p31^comet^ overexpression on normal cells, three normal cell lines, WI38, MRC-5 and BJ-T fibroblasts, were subjected to Annexin and β-gal staining. In contrast to cancer cell lines, no significant differences in Annexin V and β-gal staining between *p31* ^comet^- and empty vector-expressing cells were observed (Fig 1A and 1B). In addition, detection of floating cells was difficult after retroviral transduction, despite increased p31^comet^ expression in normal IMR90 and MRC5 cells (S1 Fig). These findings indicate that p31^comet^ overexpression does not have a significant impact on normal cells, in contrast to cancer cells.

### Regions influencing Mad2 interactions, including the C-terminus, are required for p31^comet^ -induced cell death

**Fig 2:**
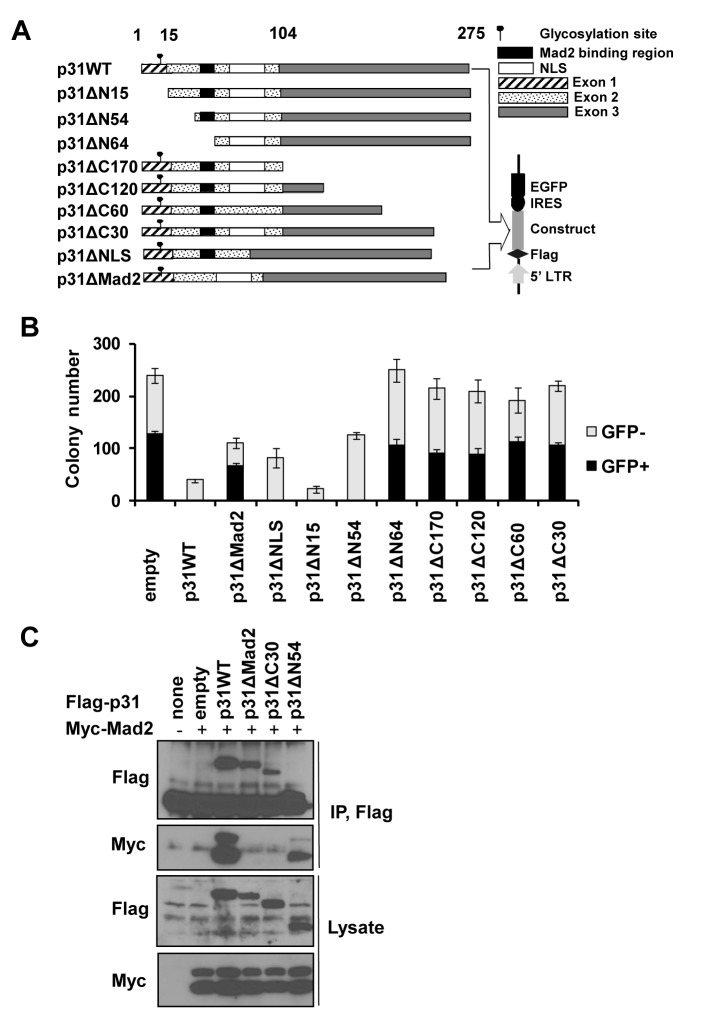
p31^comet^-induced cytotoxicity is abolished upon deletion of the C-terminus or the region affecting Mad2 binding. (A) A schematic map of human *p31* ^comet^ deletion mutants of the N- and C-termini, and regions affecting Mad2 binding and nuclear localization signal (NLS). (B) HeLa cells were infected with retroviruses containing full-length *p31* ^comet^ or the above deletion constructs and evaluated in terms of colony formation. GFP+ signifies colonies expressing p31^comet^. The total represents the sum of GFP-positive and -negative colonies. Values are presented as the mean of triplicate samples. (C) Interactions between *p31* ^*comet*^ deletion mutants and Mad2 were evaluated using an immunoprecipitation assay. Lysates from cells co-transfected with FLAG-tagged wild-type or deletion constructs of *p31* ^*comet*^ and Myc-tagged Mad2 were immunoprecipitated with an anti-FLAG antibody, and the resulting immunoprecipitates probed with an anti-Myc antibody. Expression of recombinant proteins was confirmed using antibodies directed against FLAG or Myc.]

To define the regions of p31^comet^ responsible for cell death, we generated mutants featuring sequential deletion of the N- or C-termini as well as specific regions affecting Mad2 interactions and nuclear localization (Fig 2A), and examined their effects on clonal survival of transfected HeLa cells. These constructs In contrast to cells transfected with *wt p31* ^comet^ that showed no visible GFP-positive colonies, those transfected with *p31ΔMad2* (mutant lacking Mad2 binding activity) exhibited GFP-positive colonies (Fig 2B), indicating that Mad2 binding is associated with cell death induced by p31^comet^. In this case, the deleted site belongs to the Mad2 binding region [33] but does not cover a region in contact with Mad2, based on the crystal structure of the Mad2-p31^comet^ complex [17], implying that this site influences interactions with Mad2. Unexpectedly, four deletion mutants, *p31ΔC170*, *p31ΔC120*, *p31ΔC60*, *and p31ΔC30* (lacking 170, 120, 60, and 30 residues, respectively, from the C-terminus), lost the ability to inhibit GFP-positive colony formation. In contrast, *p31ΔN15 and p31ΔN54* depleted of 15 and 54 N-terminal residues, respectively, inhibited colony formation to the same extent as full-length *p31* ^comet^, indicating that the N-terminal region does not affect cytotoxic activity. The fact that deletion of only 30 amino acids (*p31ΔC30*) completely eliminated activity clearly suggests that this C-terminal region is required for inhibition of survival. Unlike the other N-terminal deletion mutants, the *p31ΔN64* construct that lacks the region affecting Mad2 binding did not inhibit clonal survival, further supporting the association of Mad2 interactions with the suppressive effect of p31^comet^ on clonal survival. Deletion of the nuclear localization signal (*p31ΔNLS*) did not affect p31^comet^ activity. Following retroviral transduction of the above deletion constructs, p31^comet^ proteins of the appropriate sizes were expressed (S2 Fig). The results collectively indicate that the C-terminus as well as regions influencing Mad2 interactions are critical for cell death induction by p31^comet^.

As specified above, deletion of 30 amino acids from the C-terminus of *p31* ^comet^ abolished the ability to inhibit clonal survival. As the region that influences Mad2 interactions is required for this inhibition, we hypothesized that the C-terminal region is also associated with Mad2 binding. To explore this possibility, we co-transfected FLAG-tagged *p31* ^comet^ wild-type or deletion mutants together with Myc-tagged *Mad2* into HeLa cells. As expected, Mad2 protein was detected in anti-FLAG immunoprecipitates of cell lysates upon co-expression of wild-type but not mutant p31^comet^ protein (p31ΔM2) lacking Mad2 binding activity (Fig 2C). Mutant p31ΔN54 protein also co-immunoprecipitated with Mad2 protein, similar to wild-type (p31WT), indicating that N-terminal deletion of up to 54 amino acids does not affect interactions of p31^comet^ with Mad2. However, the p31ΔC30 protein did not co-immunoprecipitate with Mad2. Evidently, deletion of the C-terminal 30 residues eliminates the ability of p31^comet^ to bind Mad2. Our findings that the C-terminal region of p31^comet^ is essential for inhibition of clonal survival (Fig 2B) and interactions with Mad2 (Fig 2C) further confirm that p31^comet^ interactions with Mad2 are critical for induction of cell death.

### p31^comet^-induced cell death occurs *via* binding and inactivation of Mad2

**Fig 3:**
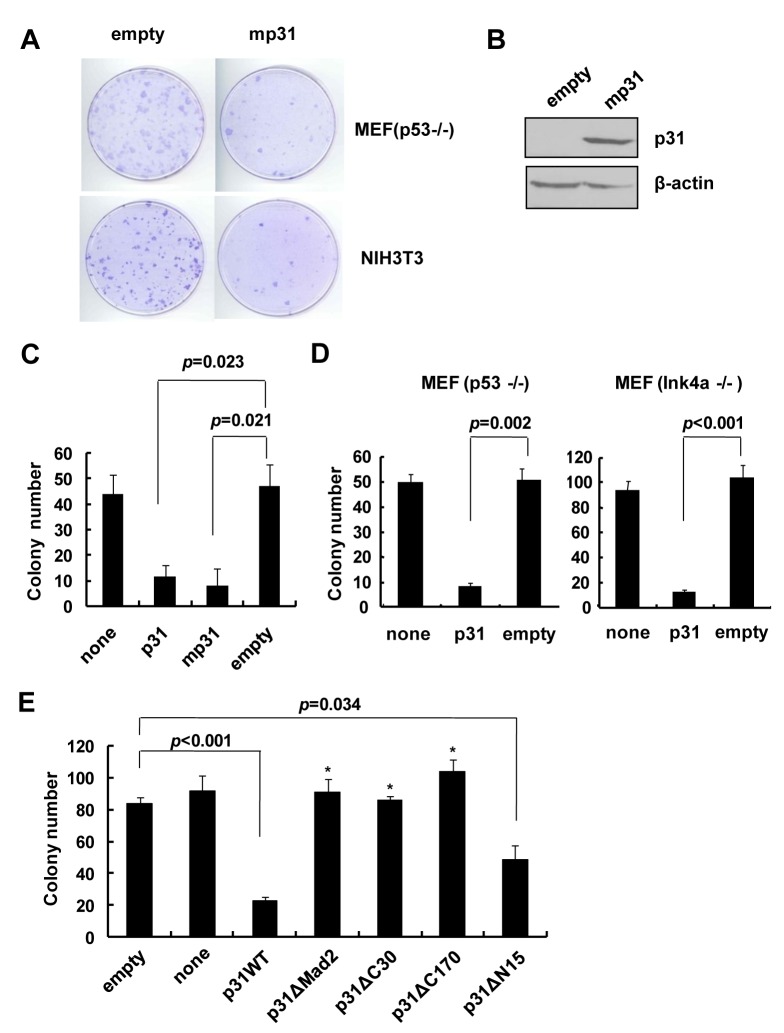
p31^comet^ inhibition of *Myc/Ras-*induced colony formation was abolished upon deletion of the region affecting Mad2 binding. (A and B) Mouse *p31* ^comet^ (*mp31* ^comet^) was co-transfected with *Myc/Ras* into *p53-/-*and NIH3T3 cells. Empty plasmid served as a control. The extent of colony formation and p31^comet^ protein level were visualized by staining with crystal violet and Western blot, respectively. (C) The abilities of human and mouse p31^comet^ to repress *Myc/Ras*-mediated colony formation in *p53-/-* MEFs were evaluated by counting the number of colonies. (D) The ability of human p31^comet^ to repress *Myc/Ras*-mediated colony formation was evaluated in *p53-/-* and *INK4a-/-* MEFs. (E) Effects of *p31* ^comet^ deletion mutants on *Myc/Ras*-mediated colony formation, compared with that of full-length *p31* ^comet^, were evaluated in *p53-/-* MEFs. Values are presented as the mean of triplicate samples. Statistical significance (*p-*value) was calculated with the Student’s t-test. * indicates *p*>0.05, where each test transfection is compared with that of empty vector.

p31^comet^-induced apoptosis or senescence began to occur 3 or 4 days after transduction and was thus examined 4 to 5 days thereafter. Under conditions where infectivity was not sufficiently high, mostly uninfected (*p31* ^comet^-untransduced) cells were present at the time of analysis, possibly leading to misinterpretation. To observe p31^comet^-induced death in association with Mad2, we used a transformed focus assay with MEF cells in which the cytotoxic activity of co-transfected genes was easily monitored by measuring colony formation [34]. As shown previously, co-transfection of *Myc* and *Ras* oncogenes resulted in the formation of dense colonies comprising transformed *p53-/-* and NIH3T3 MEF cells (Fig 3A). Upon co-transfection with a mouse homolog of human *p31* ^comet^ (designated *mp31* ^comet^), colony formation induced by *Myc/Ras* was significantly suppressed in *p53-/-*and NIH3T3 MEFs (Fig 3A and 3B). Reduction in colony formation was also achieved when human *p31* ^comet^ was co-transfected with *Myc/Ras* in NIH3T3 cells (Fig 3C). As with *p53-/-* MEFs, *p31* ^comet^ repressed colony formation of *INK4a-/-* MEFs (Fig 3D). Thus, evaluation of p31^comet^ cytotoxicity was possible *via* assessment of colony formation upon *Myc/Ras* co-transfection. Using the transformed focus assay, we re-examined the effects of specific deletions on p31^comet^-induced death. Similar to the results with human cancer cell lines (Fig 2B), *p31ΔMad2* and two C-terminal deletion mutants, *p31ΔC170* and p31ΔC30, defective in Mad2 binding did not inhibit *Myc/Ras*-induced colony formation, in contrast to wild-type *p31* ^comet^, indicating loss of inhibitory activity (Fig 3E). However, the N-terminal deletion mutant, *p31ΔN15*, that did not affect Mad2 interactions displayed inhibitory activity, similar to its wild-type counterpart. The finding that deletion of only 30 C-terminal residues (*p31ΔC30*) completely eliminates activity further highlights the importance of this region in inhibition of clonal survival. Our results confirmed that regions affecting Mad2 interactions, including the C-terminus, are essential for p31^comet^-overexpression induced cell death. This finding, together with data showing that the C-terminal region is required for Mad2 binding (Fig 2C), support an essential role of Mad2 in cancer cell death induction by p31^comet^.

**Fig 4:**
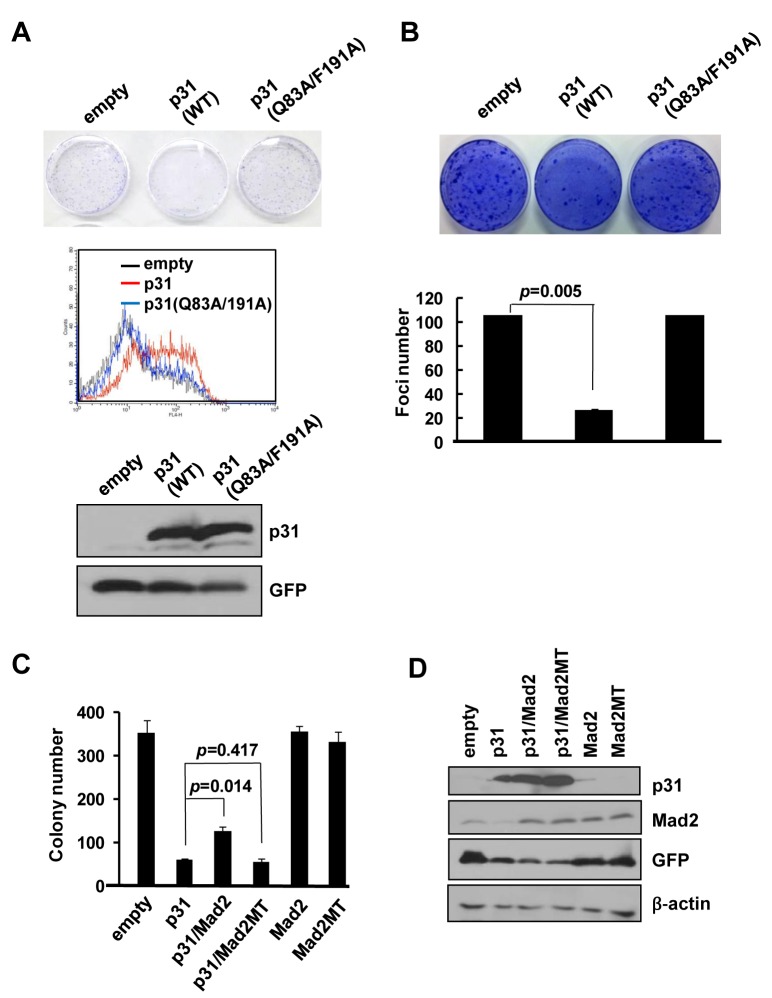
Mad2 binding activity is essential for p31^comet^ inhibition of colony formation. (A) Effects of a *p31* ^*comet*^ mutant (*Q83A/F191A*) on apoptosis (lower panel) and repression of colony formation (upper panel) were analyzed in HeLa cells. Expression of p31^comet^ and Q83A/F191A was confirmed *via* Western blot. (B) The effects of a p31^comet^ mutant (*Q83/F191A*) on *Myc/Ras*-mediated colony formation in p53-/- MEFs were compared with those of wild-type (WT) *p31* ^comet^. (C) The effect of *Mad2* on p31^comet^-induced repression of clonogenic cell survival was analyzed in HeLa cells in the presence of either wild-type or dominant-negative mutant Mad2 (*R133E/Q134A*) (Mad2mt). (D) Expression of p31^comet^, Mad2, and R133E/Q134A mutant (Mad2mt) was confirmed using immunoblotting. Values represent mean of triplicate samples. Statistical significance (*p-*value) was calculated with Student’s t-test. * indicates *p*>0.05, when each test transfection was compared with that of empty vector.

To provide further evidence that p31^comet^-induced death relies on Mad2 interactions, we examined a *p31* ^comet^ (*Q83A/F191A*) mutant lacking binding activity to Mad2 [17]. In contrast to wild-type *p31* ^*comet*^, the mutant form did not suppress clonal survival when transduced into HeLa cells *via* retroviral infection (Fig 4A) or MEFs with Myc/Ras co-transfection (Fig 4B). Consistently, HeLa cells infected with the *Q83A/F191A* mutant were not susceptible to Annexin staining, in contrast to those infected with wild-type *p31* ^comet^ (Fig 4A). These findings imply that p31^comet^-induced death occurs only when Mad2 binding activity is retained, ultimately leading to inhibition of Mad2 action. To explore this point, we performed an experiment to establish whether p31^comet^-induced death can be rescued by Mad2. To this end, HeLa cells retrovirally infected with *p31* ^comet^ were reinfected with retroviruses containing wild-type *Mad2*. As expected, p31^comet^ -induced suppression of clonal survival was reversed by wild-type *Mad2* (Fig 4C and 4D). However, the dominant-negative form of *Mad2 (R133E/Q134A)* lacking the ability to bind to p31^comet^ (and devoid of spindle checkpoint activity) [21] did not affect survival (Fig 4C and 4D). *Mad2* or its mutant (*R133E/Q134A*) alone did not suppress colony formation. These data support the theory that Mad2 inactivation is essential for cell death induction by p31^comet^. Furthermore, the balance between p31^comet^ and Mad2 levels may determine the cytotoxic effect.

### p31^comet^-induced cell death accompanies spindle checkpoint inactivation in a similar manner as Mad2 depletion

**Fig 5:**
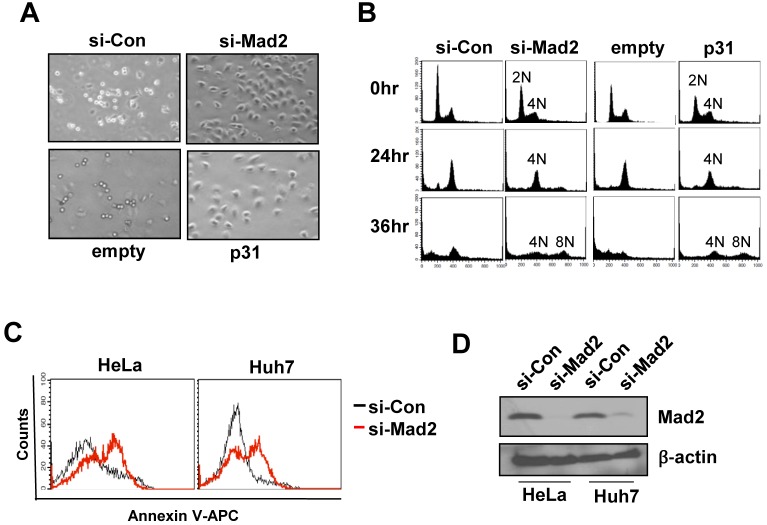
p31^comet^ overexpression leads to mitotic failure similar to Mad2 depletion. (A) Nocodazole was added to cultures of HeLa cells transfected with either *Mad2* siRNA (si-Mad2) or control siRNA (si-Con) and transduced retrovirally with *p31* ^comet^ or empty vector. Subsequent morphological changes were compared under a light microscope (x100). (B) Under the above conditions, cell cycle progression was monitored with histograms of the DNA content (2N, 4N and 8N) at 24 h and 36 h after treatment. (C) Apoptosis was analyzed *via* Annexin V staining in HeLa and Huh7 cells transfected with either *Mad2* siRNA (si-Mad2) or control siRNA (si-Con). (D) Mad2 depletion with *Mad2* siRNA was confirmed using Western blot.

Our previous report showed that p31^comet^ overexpression leads to massive chromosomal aberration, including the formation of anaphase bridge, micronuclei and multinuclei [24], similar phenomena to those observed upon depletion of Mad2 [35, 36]. In view of the collective results showing that p31^comet^ overexpression leads to cell death in association with Mad2 inactivation, we hypothesized that Mad2 depletion should yield a similar phenotype to p31^comet^ overexpression. To ascertain this theory, we examined the defects in checkpoint function after the addition of nocodazole, a microtubule destabilizer, under both conditions (p31^comet^ overexpression and Mad2 depletion). In HeLa cells transduced with control siRNA or infected with control retrovirus, nocodazole treatment led to acquisition of a rounded cell shape (Fig 5A), followed by transient arrest of the cell cycle at the G2/M phase and accumulation of cells in the sub-G0/G1 phase after 36 h (Fig 5B). Upon Mad2 depletion or p31^comet^ overexpression, cell shape change and sub-G0/G1 occurrence were inhibited and aneuploidy observed (Fig 5B), indicating inactivation of spindle checkpoint function. In addition, Mad2 depletion led to an increase in the number of cells positive for Annexin V (Fig 5C and 5D), analogous to p31^comet^ overexpression (Fig 1A), indicating that apoptotic death occurs similarly under both conditions. We previously reported senescence in the same cell line *via* either Mad2 depletion or p31^comet^ overexpression [24]. The collective previous and present data showing that p31^comet^ overexpression leads to similar phenotypes as Mad2 depletion further support the theory that p31^comet^-induced apoptosis and senescence occur *via* binding and inactivation of Mad2.

## Discussion

Regulators of the mitotic cell cycle, including spindle checkpoint, have emerged as important targets in cancer therapy, and several inhibitors of these proteins have been developed to date [37]. Mad2 is considered a useful marker for anticancer therapy [10–15]. Cancers with Rb deletion overexpress Mad2 owing to E2F activation, which is associated with poorer prognosis in patients [10]. Constitutive Mad2 overexpression has been shown to result in chromosomal instability that can enhance cancer aggressiveness and reduce response to chemotherapy [15, 23]. Thus, modulation of aberrant Mad2 expression may provide a useful strategy in cancer treatment.

In the present study, we showed that p31^comet^ overexpression leads to apoptosis and senescence in a broad range of human cancer cells, achieved through interactions with Mad2 and inhibition of its function. Structural analysis using deletion mutants of p31^comet^ revealed that the C-terminal region is required for Mad2 interactions. Consistent with previous findings [24], p31^comet^ overexpression resulted in inhibition of clonal survival, as evident from analysis of colony formation. Notably, deletion of the region affecting Mad2 binding abolished p31^comet^-induced inhibition of clonal survival. Mad2 binding activity was eliminated following deletion of 30 amino acids from the C-terminus of *p31* ^comet^ (Fig 2) or introduction of mutations at two distinct sites (*Q83A/F191A*) [17]. The C-terminal deletion and point mutations additionally abolished the cytotoxic activity of p31^comet^. In contrast, N-terminal deletion of up to 54 amino acids affected neither Mad2 binding nor cytotoxic activity of p31^comet^. However, extended deletion of up to 64 amino acids from the N-terminus (leading to elimination of the region affecting Mad2 interaction) abolished p31^comet^ activity. These findings suggest that Mad2 interactions are essential for p31^comet^-induced cell death. Based on a recent report showing that phosphorylation inhibits p31^comet^ interaction with Mad2 [38], modifications in phosphorylation are proposed to control cytotoxicity in association with Mad2 interaction.

In the present study, the majority of cancer cell lines tested exhibited both phenotypes while only a few cell lines displayed senescence upon retroviral transduction of p31^comet^. Exogenously increased amounts of p31^comet^ were similar between the two phenotypically different cell lines (Fig 1 and S1 Fig). For example, A549 and H1299 cell lines primarily undergoing senescence exhibited similar levels of exogenous p31^comet^ protein, compared with HeLa, Huh7, and Chang liver cells with both phenotypes. Endogenous levels of p31^comet^ protein were also similar in all the cell lines employed (S1 Fig). Our findings clearly suggest that the differential responses are not simply attributable to variations in p31^comet^ protein levels.

p31^comet^ overexpression leads to cell death that disrupts the spindle checkpoint. The ability of p31^comet^ to inhibit clonal survival is attributable to interactions with Mad2 and consequent inactivation. Indeed, complete depletion of Mad2 induces mitotic defects by rendering the APC-Cdc20 complex free of the protein, eventually causing apoptosis and senescence [35, 39], similar to data obtained with p31^comet^ overexpression [24]. However, a partial decrease in Mad2 expression, evident when only one copy of *Mad2* is deleted, results in premature anaphase and chromosomal instability but without loss of cell viability [40]. Therefore, the inhibitory activity of p31^comet^ on clonal survival appears to be mediated by complete inhibition of Mad2. p31^comet^ may be the most powerful tool for inhibiting Mad2, and its expression may serve to control excessive Mad2 activity frequently observed in human cancers. Consistent with this theory, p31^comet^ exerted inhibitory activity on survival in a broad range of human cancer cells.

Our earlier observation that p31^comet^-induced senescence is affected by p21WAF1/CIF1 and p53 was based on A549 cells, which primarily undergo senescence but rarely apoptosis. Owing to the paucity of data showing a direct link of the spindle checkpoint to cellular senescence, our prior report focused on defining the relationship between abnormal checkpoint and p31^comet^-induced senescence in susceptible A549 cells. We showed that p31^comet^-induced senescence is partially inhibited by depletion of p53 and completely by depletion of p21WAF1/CIF1. However, data from the present study showed that p31^comet^ -induced senescence occurs in both p53-defective and -proficient cells. Therefore, our earlier finding that p31^comet^-induced senescence is dependent on p53 and p21WAF1/CIP1 appears to be valid only in p53-proficient A549 cells that primarily undergo senescence.

Mad2 is a core component of the spindle checkpoint that controls the fidelity of chromosome segregation [41, 42]. Reduction in Mad2 levels generates chromosomal instability by disrupting normal chromosomal segregation [40]. Previously, we showed that overexpression of p31^comet^ causes aberrant segregation accompanied by the development of an imbalance in chromosome number. Additionally, p31^comet^ induces chromosomal abnormalities, as evident from the development of anaphase bridges and micronuclei [24]. Thus, inhibition of clonal survival by p31^comet^ is attributable to malfunction of the spindle checkpoint as a consequence of direct binding and inactivation of Mad2. Utilization of p31^comet^ to regulate Mad2 activity may thus present a valuable therapeutic strategy for cancer. Moreover, the recent finding that p31^comet^ depletion increases sensitivity to antimitotic drugs further supports its potential in cancer therapy [43].

## Conclusions

The regions of p31^comet^ that affect Mad2 interactions, including the C-terminus, are critical for induction of cell death. This cytotoxicity of p31^comet^ is mediated through direct binding and inactivation of Mad2.

## Supporting Information

S1 Fig Levels of p31^comet^ after retroviral transduction. Western blot analysis of p31^comet^ and GFP protein expression in various cancer and normal cell lines retrovirally infected with *p31* ^*comet*^ and control empty vector.
(PDF)

S2 Fig Protein expression of p31^comet^ deletion mutants. HeLa cells were retrovirally infected with wild-type *p31* ^*comet*^ (*p31* ^*comet*^ WT) and deletion mutants, and protein expression analyzed via western blot.
(PDF)
