# Supplementary figures and images for: p31comet-Induced Cell Death Is Mediated by Binding and Inactivation of Mad2
Source: PLoS One. 2015 Nov 6;10(11):e0141523. doi: 10.1371/journal.pone.0141523 (PMC4636321; doi:10.1371/journal.pone.0141523)

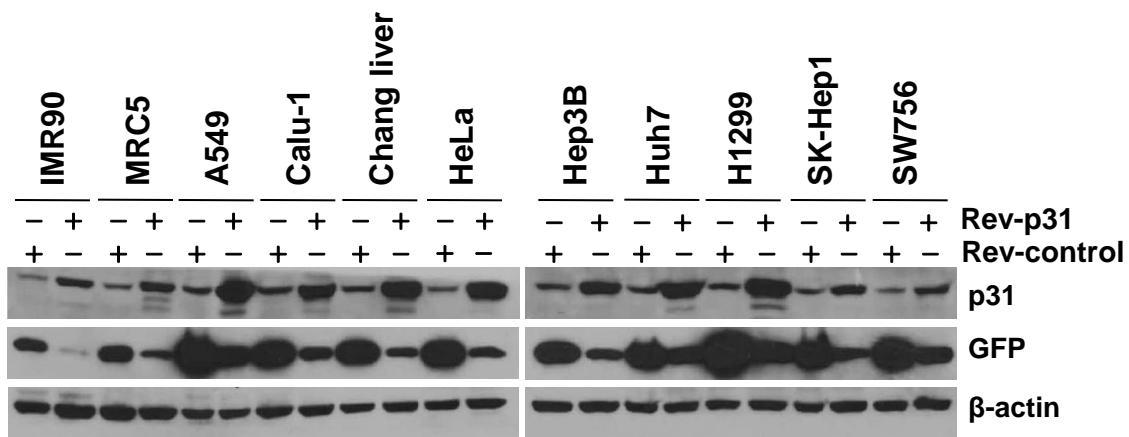

**S1 Fig. Levels of p31<sup>comet</sup> after retroviral transduction**

Supplement: S1 Fig — Western blot analysis of p31comet and GFP protein expression in various cancer and normal cell lines retrovirally infected with p31 comet and control empty vector. (PDF) [file pone.0141523.s001.pdf]

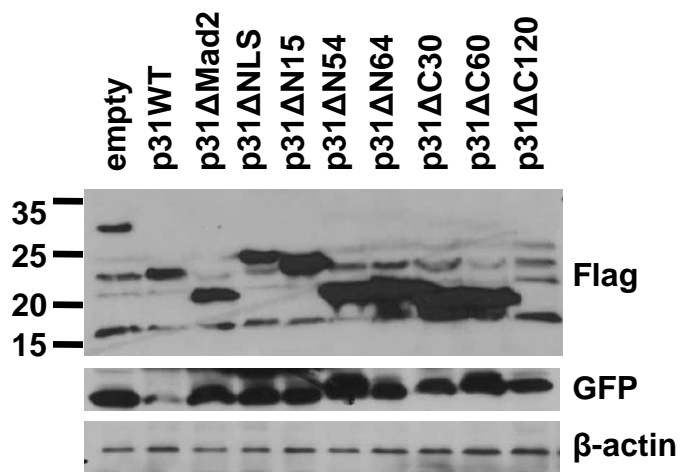

**S2 Fig. Protein expression of p31<sup>comet</sup> deletion mutants**

Supplement: S2 Fig — HeLa cells were retrovirally infected with wild-type p31 comet (p31 comet WT) and deletion mutants, and protein expression analyzed via western blot. (PDF) [file pone.0141523.s002.pdf]
